# High throughput screening system for engineered cardiac tissues

**DOI:** 10.3389/fbioe.2023.1177688

**Published:** 2023-05-11

**Authors:** Marshall S. Ma, Subramanian Sundaram, Lihua Lou, Arvind Agarwal, Christopher S. Chen, Thomas G. Bifano

**Affiliations:** ^1^ Mechanical Engineering, Boston University, Boston, MA, United States; ^2^ Photonics Center, Boston University, Boston, MA, United States; ^3^ Biomedical Engineering, Boston University, Boston, MA, United States; ^4^ Mechanical and Materials Engineering, Florida International University, Miami, FL, United States

**Keywords:** high throughput imaging(HTI), engineered cardiac tissue, cardiotoxicity screening, PDMS, micro manufacturing

## Abstract

**Introduction:** Three dimensional engineered cardiac tissues (3D ECTs) have become indispensable as *in vitro* models to assess drug cardiotoxicity, a leading cause of failure in pharmaceutical development. A current bottleneck is the relatively low throughput of assays that measure spontaneous contractile forces exerted by millimeter-scale ECTs typically recorded through precise optical measurement of deflection of the polymer scaffolds that support them. The required resolution and speed limit the field of view to at most a few ECTs at a time using conventional imaging.

**Methods:** To balance the inherent tradeoff among imaging resolution, field of view and speed, an innovative mosaic imaging system was designed, built, and validated to sense contractile force of 3D ECTs seeded on a 96-well plate. Results: The system performance was validated through real-time, parallel contractile force monitoring for up to 3 weeks. Pilot drug testing was conducted using isoproterenol.

**Discussion:** The described tool increases contractile force sensing throughput to 96 samples per measurement; significantly reduces cost, time and labor needed for preclinical cardiotoxicity assay using 3D ECT. More broadly, our mosaicking approach is a general way to scale up image-based screening in multi-well formats.

## 1 Introduction

During late-stage drug development, cardiovascular toxicity is the leading cause and most severe form of adverse drug reactions (ADRs) that contribute to nearly one-thirds of drug attritions, including post-approval withdrawals (Kurokawa and George., 2016; Helmy et al., 2017; Tsao et al., 2022). Cardiotoxicity screening requires large testing populations and long-term chronic monitoring: under the current paradigm, a single compound may take approximately 12 years and US $2 billion to be declared fully safe for clinical use (Mann, 2017; Moore et al., 2018; Pang et al., 2019). There is a strong need for high throughput assays based on engineered tissue models with reliable predictivity. Over the past decades, engineered cardiac tissues (ECTs) made with human induced pluripotent stem cell-derived cardiomyocytes (hiPSC-CMs) have become powerful model systems with proven physiological similarity (Greenberg et al., 2018; Portillo-Lara et al., 2019; Savoji et al., 2019; Schwach et al., 2020). However, functional characterization of the ECTs is still low throughput, preventing ECTs from being used in standard cardiotoxicity screening (Li et al., 2015; Gintant et al., 2020).

There is an emerging consensus in the field that 3D ECTs provide better predictivity for cardiotoxicity screening as compared to rodent models because they more closely approximate human cardiac tissues and because they allow easier measurements of cardiac function including contractility (Herron et al., 2016; Rocha et al., 2017; Ronaldson-Bouchard et al., 2018). Previous studies focused on accelerating electrophysiological measurements, such as the action potential of the cardiomyocytes and the Ca2+ transient for ECTs in the 2D format (Sharma et al., 2018; Gintant et al., 2019; 2020; Savoji et al., 2019). But the scale and throughput of these 2D assays cannot be transferred to 3D ECT-based assays due to the complexity of scaffold fabrication and optical imaging.

A commonly used architecture for3D ECTs is comprised of 1–2 mm long bundles of cohesive tissue suspended between two compliant polydimethylsiloxane (PDMS) micro-pillars. These structures, namely, micro tissue gauges (μTUGs), were initially developed by the Chen group at Boston University (Legant et al., 2009) inspired by early models built with embryonic cardiomyocytes (Eschenhagen et al., 1997). With a uniaxial bending model for the pair of upright micropillars, contractile force in a μTUG can be determined by microscopic imaging of pillar deflection, then relating this deflection to force through a known pillar stiffness and an assumption of linear elasticity for the pillar. Lateral deflections at the top of each pillar in response to contractile ECT twitches range from a few micrometers to a few tens of micrometers. To date, most ECT contractility measurement systems that use optical imaging to infer twitch force are limited in throughput: they can only measure a small number of ECTs simultaneously. To monitor ECT contractility in an industry-standard 96 well-plate format, for example, one must currently rely on sequential imaging using robotic stage control. While some studies have demonstrated progress toward higher throughput assays (Legant et al., 2009; Hansen et al., 2010; Spreeuwel et al., 2014; Polacheck and Chen, 2016; Mannhardt et al., 2017; Rocha et al., 2017; Martins et al., 2021), a bottleneck has been the field of view (FOV), and most previous efforts have relied on low-throughput serial imaging of individual wells or small groups of wells.

The main engineering challenge in high-throughput contractile force monitoring of ECTs is that the fundamental optical measurement technique used to assess that force is constrained by an inherent optical tradeoff among three characteristics of the optical system: imaging resolution, imaging field of view (FOV), and imaging speed or frame rate. To adapt conventional ECT contractile force monitoring to the industry-standard 96-well plate format would require an imaging system with ∼10 µm resolution, 120 mm field of view, and 60 Hz imaging frame rate.

In our work, we have overcome this barrier through the development of an innovative optical mosaic imaging system supplemented by a 96 well molding process to allow 3D ECT scaffolding, culture, and evaluation in the standard 96-well plate format. To validate the practical use of our system in a standard cardiotoxicity screening workflow, the following challenges were addressed through experiments: have adequate resolving power over the FOV of a 96-well plate; have enough processing bandwidth to sense compound-induced responses from 96 ECTs; be able to continuously monitor contractile force over long period of ECT culture.

## 2 Materials and methods

### 2.1 Optical setup

The main optical innovation in the system we developed was inspired by the observation that a conventional image of the entire field of view of a 96 well plate contains useful information only in a small subset of the imaged pixels. Since area of each well is 81 mm^2^ and the area occupied by an individual µTug is ∼9 mm^2^, in a conventional image almost 90% of the pixels are not used for the contractility measurement. By inserting a lens array between the well-plate and a conventional telecentric imaging system, we were able to magnify the regions of interest, producing a mosaic image on the camera in which most pixels were associated with the µTug. This efficient increase in resolution without compromising field of view allowed us to achieve full 96 well imaging at the required frame rate, field of view, and resolution using off-the-shelf commercial hardware.

To meet the required temporal resolution of sensing ECT contraction, a standard CMOS sensor with gigabit ethernet connection (ORX-10G-123S6M-C, FLIR) was selected to allow image acquisition and data transfer at 60 Hz on a pitch of 4096 × 3000 pixels (3.45 µm/pixel). A bi-telecentric lens (MTL-15018C-012, Moritex) was used to project the 80 mm × 120 mm FOV for a 96-well plate to the camera sensor. The ROI within each well was then individually magnified by 96 achromatic doublet lenses (AC080-016-D8-UC-SP, FL = 26mm, ∅ = 8.4 mm, Thorlabs) held in a custom “fly-eye” lens array. The layout of the lens array matched the layout of standard 96-well plate (9 mm pitch between neighboring well): each lens was concentrically aligned with center of each well on the 96-well plate.

All optical components were vertically placed for the ease of loading and unloading ECT samples to the sample stage. To allow easy adjustment of their location, optics were attached to an optical rail (XT95, Thorlabs). The lens array was inserted between the object plane (sample stage) and the telecentric lens, at a location where the resultant intermediate image plane is a working distance (300 mm) away from the telecentric lens. Given the focal length of the lens array (f_la_ = 26 mm), the position of the lens array (*S*_*img*_) and the position of the sample stage (*S*_*obj*_) for any desired lens array magnification (*M*_*la*_) are governed by the following equations:

$$\frac{1}{S_{obj}}+\frac{1}{S_{img}}=\frac{1}{f_{la}}$$ (1)

$$\frac{S_{img}}{S_{obj}}=M_{la}$$ (2)

By changing the axial placement of the lens array, we are able to vary the magnification of the mosaic image over a practical range from 3× to 6×.

**FIGURE 1:**
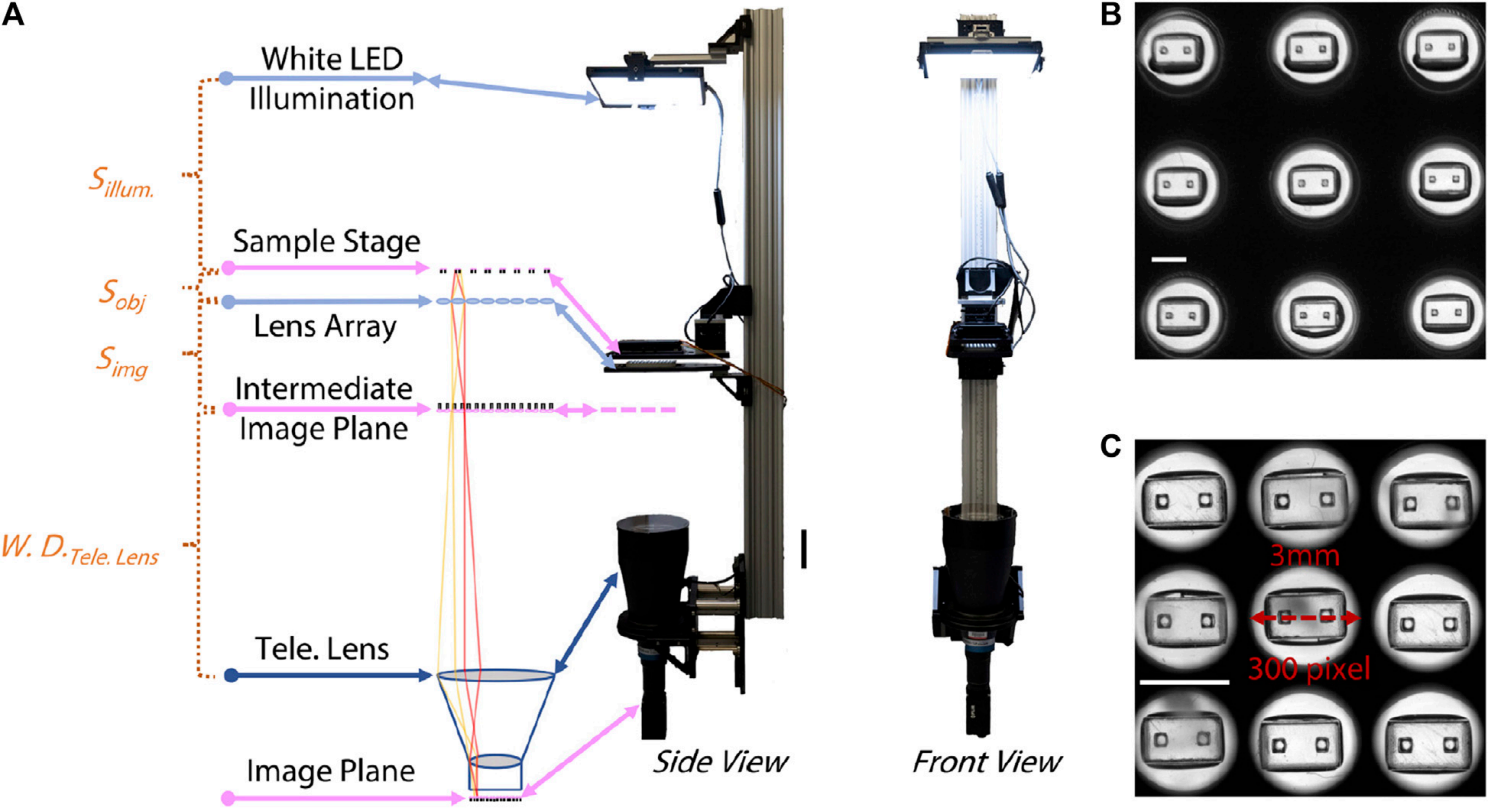
Optical setup of the “mosaic” imaging system. **(A)** Left to right: sketch of the optical pathway, side view and front view of the system. Distances between optics are labelled referring to Eqns [1] and [2]. Scale bar is 20 cm. **(B)** Image of 3 × 3 empty µTUGs segmented from a snapshot taken without the lens array. Scale bar is 2 mm. **(C)** Image of the same 3 × 3 empty µTUGs segmented from a snapshot taken with the lens array. Scale bar is 2 mm.

A white LED panel (BL1010-WHI24, Advanced Illumination) provided wide-field, diffused illumination to the optics, with nominal output of 225W/m^2^. A stage-top incubator (UNO-T-H-CO2/H101-K, Okolab) was installed at the sample stage to maintain the physiological environment for the ECT (37 ± 0.5°C temperature, 95% ± 1% CO_2_ concentration and ≥80% humidity). The assembly of this inverted microscopic setup is shown in Figure 1A, except for the custom lens array (see Supplementary Material for details), all other components are commercial, off-the-shelf products. The effect of the lens array is illustrated in Figure 1B, C by imaging empty µTUGs that have individual ROIs that are 2 mm wide and 1 mm tall. Without the lens array, the mosaic of ROI only occupied <50% pixels in the resultant image, while the lens array increased the effective use of the FOV by magnifying individual ROI by 3×.

### 2.2 Parallel molding of the µTUGs

**FIGURE 2:**
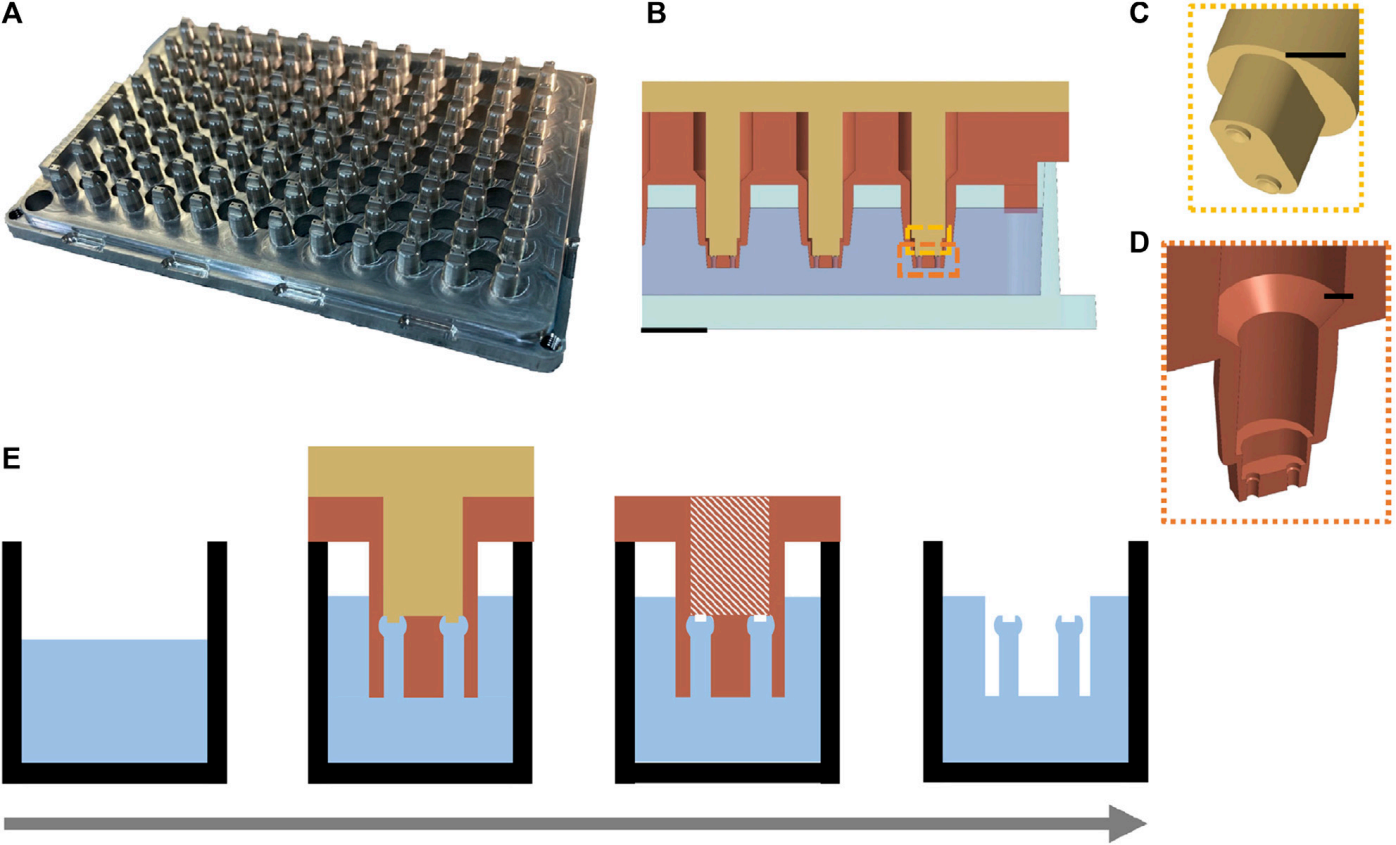
Design of a parallel process to mold 96 µTUGs in a standard wellplate. **(A)** Isometric view of the aluminum parallel stamping mold. **(B)** Cross-sectional view of the mold-wellplate assembly during the molding process. Scale bar is 2 mm. **(C)** Exploded view of the comb (top piece) that creates the release-aiding notches on the caps of the µTUGs. Scale bar is 1 mm. **(D)** Exploded view of the sleeves (bottom piece) that contains the inverse structure of the µTUGs. Scale bar is 1 mm. **(E)** key steps in the molding procedure modified from the traditional casting and molding process. Left to right: substrate preparation and casting, mold filling, comb releasing and sleeve releasing.

Based on the serial casting and molding process for µTUGs used in previous practice, our optimization focused on reducing the repetitive pick-and-place of the individual stamping molds. A 2-piece aluminum mold with an array of 96 stamping molds was designed (Figure 2A), see Supplementary Material for details). Each stamp contained the inverse structure of a single uTug (Figure 2B). The comb (top piece colored in gold) created a pair of notches on the cap of the micropillars to aid mold release and to introduce high-contrast target feature for imaging (Figure 2C). The comb could slide in and out of the sleeve (bottom piece colored in sienna) smoothly. During molding, set screws were tightened to ensure proper alignment of the comb and sleeve. Instead of releasing individual molds 96 times, this parallel mold allowed releasing to be done in a 2-step operation: lifting of the comb and the sleeve (Figure 2D).

A single well plate (75999-254, VWR) with the same dimension as the 96-well plate was prepared as substrate. 58 g of 10:1 (base to curing agent) PDMS mixture (Sylgard 184, Corning) was poured into the substrate. After cleaning and drying, the aluminum mold was assembled with the PDMS containing wellplate, step edge of the mold was fitted with the inner edge of the substrate plate. Vacuum degassing was performed between each step. The assembly was placed in level at room temperature (∼20–25°C) for 40–48 h to cure. After curing, the comb is released by turning the set screws against the sleeve. Then, the sleeve is released by turning the set screws against the wellplate corner. Two arrays of set screws that turn against the sleeve and the wellplate corner provided controlled lifting of the comb and the sleeve during demolding. The notches on the top of the µTUGs aided the deformation of the caps so that they could be squeezed out of the mold easier. As a result, this procedure does not require releasing agents. An overview of the molding procedure is illustrated in Figure 2E. Once finished, a conventional microscope (20×, BX51, Olympus) was used to inspect the manufacturing quality by checking the structural integrity of the µTUGs.

### 2.3 Mechanical modeling of the micropillar

During the spontaneous contraction of the ECTs, the deflection of the micropillars is due to a combination of bending, shear, and distortion of the pillar’s attachment to the scaffold base. Even though the pillars are not rigidly fixed because of the compliant PDMS foundation, the lateral stiffness of the pillars can be assumed to be constant as the material is linearly elastic (Mannhardt et al., 2016; Ma et al., 2019). The relation between the contractile force exerted on one of the pillars (*F*_*1*_) and the deflection of this pillar (*δ*) can be represented below, where (*k*) is the lateral stiffness of the pillar:

$$F_{1}=k\;\delta$$ (3)

The overall lateral deformation of the ECT is the sum of the lateral deflections of both pillars. Assuming the pillars are geometrically symmetric, the lateral deflection on each pillar is equal. With the experimentally measured micropillar stiffness (*k*_*exp*_), the contractile force of the ECT (*F*_*ECT*_) can be calculated from the imaged lateral micropillar deflection (*δ*):

$$F_{ECT}=k_{\exp}\;\delta$$ (4)

### 2.4 Measurement of micropillar stiffness

To experimentally measure the stiffness of the molded micropillars, a nanoindenter (Hysitron Biosoft, Bruker) was employed using a conospherical probe with a radius of 50 µm. Height of the contact point was defined by the anchor point of the ECT on the pillar. Measurement was conducted using displacement-control mode with µTUGs placed on a glass slide. Indentation cycle included loading (5 μm/s for 100 µm) and unloading (−5 μm/s for −100 µm) intervals. An endoscope camera recorded the process to capture initial indenter-µTUG contact, indentation location, and indentation process (see videos in Supplementary Material). Stiffness was calculated as the slope of the initial unloading section in the load-displacement curves using a linear least squares fit. Eight to ten tests were conducted for µTUGs with varying shapes, pre-seeding curing time, post-seeding curing time, and post-treatment methods. Two-sided significant testing was applied to calculate differences among the measured lateral stiffnesses of those µTUGs.

### 2.5 3D ECT tissue culture

To prepare the 96-well plate for seeding, the wells were subject to functionalization steps to ensure that the tops of the PDMS micropillars were selectively adhesive for the final self-assembled tissue. First, the 96-well plate with the µTUGs was treated with air plasma for 30 s at 100 W (EMS1050X, Electron Microscopy Sciences). Each well was then treated with 0.01% poly-l-lysine for 2 h (diluted in DI water from 0.1% poly-L-lysine, MilliporeSigma) so that the tops of the micropillars and the inner well are fully covered. Subsequently, after aspiration and rinsing with PBS, 1% glutaraldehyde was added to each inner well covering the tops of the micropillars and stored at room temperature for 10 min. The wells were rinsed 3 times and then covered with DI water and left overnight at 4°C. On the day of cell seeding, the wells were sterilized under UV for 15 min. Finally, to ensure the cells and tissue do not adhere to the bottom of the inner well, the bottom surfaces were treated with 2% Pluronic F-127.

After treatment of the wells, compacted tissues were formed around the PDMS micropillars in each well through self-assembly as described previously (Boudou et al., 2012), from an initial cell-laden gel filling the rectangular inner well containing 54,000 iPSC-derived cardiomyocytes and 6,000 stromal cells (human mesenchymal stem cells, hMSCs). The iPSC-derived cardiomyocytes (iPSC-CMs) used in our study were differentiated from the PGP1 line following a protocol based on the temporal regulation of the Wnt signaling pathway (Lian et al., 2012) and purified by lactate starvation for 4 days (in glucose-free RPMI supplemented with 4 mM sodium lactate). Upon completion of the differentiation protocol, these iPSC-CMs were replated on fibronectin-coated dishes and cultured for over 2 weeks (in RPMI with B-27 supplement). A total of 60,000 cells was added to each well, suspended in 6 µL of extracellular matrix comprising of Fibrin (final concentration of 4 mg/mL using human fibrinogen and 0.4U/mg Thrombin, MilliporeSigma) and 10% Matrigel (Corning) mixed in base media (RPMI with Gibco B-27 supplement, Aprotinin and ROCK inhibitor Y-27632). To prevent the clotting of fibrinogen prior to seeding into the 96 wells, first, 2 µL of base media containing the total amount of thrombin was added to each well and spread uniformly across the bottom of the inner well. Then, 4 µL of the solution containing the 60,000 cells and required ECM (Matrigel and Fibrinogen) were added to the inner well and mixed once with the pipette. Every two adjacent columns were sealed with parafilm strips to limit evaporation and the plates were inverted immediately following the addition of cells to allow the cells to settle near the tops of the PDMS micropillars as the Fibrin clots. Immediately after the clotting of Fibrin (∼5min), the plates were flipped back and 100 µL of growth media (DMEM with 10% fetal bovine serum, 1% Nonessential amino acids, 1% GlutaMAX and 1% penicillin-streptomycin) was added to each well. The growth media used after seeding was supplemented with 5 µM Y-27632 and 33 μg/mL of aprotinin. Y-27632 was removed after 1 day of culture, and the concentration of aprotinin was halved after a week. The 96 well plates were stored in a tissue culture incubator at 37°C with 5% CO2 and the media was replaced every 48 h. Over a period of 2–5 days, the microtissue detaches from the edges of the inner well and compacts around the tops of the two PDMS pillars.

### 2.6 Micropillar tracking and deflection conversion

A block-matching program was implemented in MATLAB to obtain micropillar deflection by recording the time series of the selected ROI within the specified measurement period. Before tracking started, the centers of the correlation window were manually selected on a snapshot (reference frame for the correlation) of the entire wellplate. Typically, features with high contrast such as the notches on the cap of the µTUGs were selected to maximize imaging quality. 96 correlation windows were generated with width and height set for the configured optical setting (overall magnification of the optics). MATLAB then continuously tracks the target feature in each correlation window. Parallel computing must be enabled to allow larger processing bandwidth than video acquisition. Length of each measurement video was controlled at 2000 frames (∼30 s). Deflection of the micropillar was obtained by averaging the peaks on the selected segments of the displacement-time plot. To convert the imaged deflection in pixels to microns, an optical ruler (R1L3S2P, Thorlabs) with precise line gratings of known lengths was imaged.

## 3 Results

### 3.1 Resolution validation of the optical system

In prior work, the average peak contractile force for this geometry of µTug was about ∼100 µN and the average lateral micropillar stiffness was measured to be ∼5 μN/μm (Legant et al., 2009; Hansen et al., 2010; Boudou et al., 2012; Spreeuwel et al., 2014; Polacheck and Chen, 2016; Mannhardt et al., 2017; Rocha et al., 2017; Thavandiran et al., 2020; Martins et al., 2021; Li et al., 2023). In the system reported here, the imaging resolution limit was about 10 μm, micropillar localization precision was 1 µm, and lateral stiffness of µTUG micropillars was averaged 5.3 μN/μm. The resultant nominal sensitivity of contractile amplitude for the described parallel high throughput imaging system is ∼0.2 µN.

**FIGURE 3:**
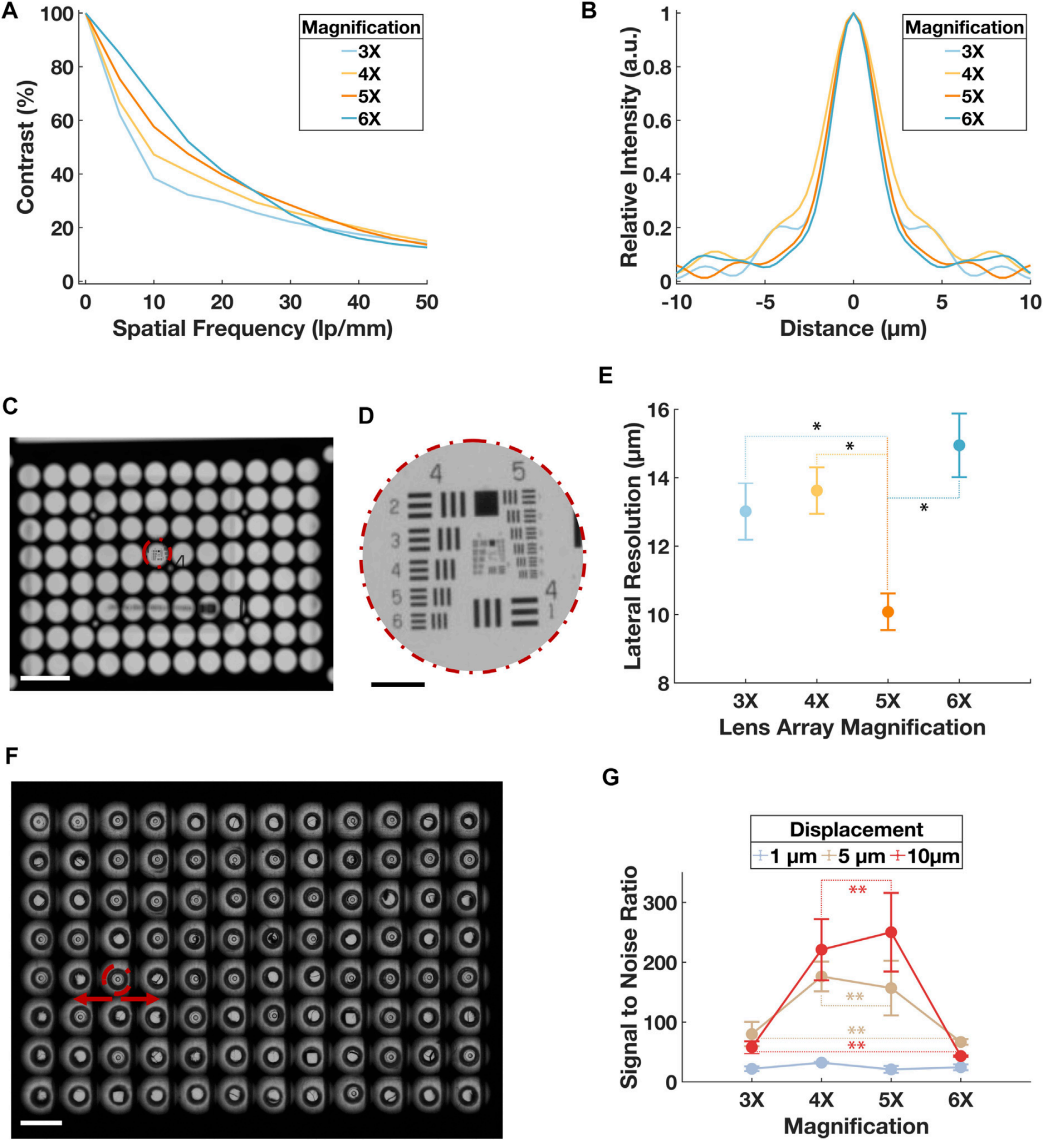
Validation of the optical resolution of the system. **(A)** Simulated MTF in terms of contrast in percentage and special frequency in lp/mm for the optical system when lens array magnification was at 3×, 4×, 5× and 6×. Point source with 3 distributed wavelengths covering the entire visible spectrum was used to represent the practical illumination. **(B)** Simulated PSF in terms of the relative intensity and distance from the central axis. Point source with 3 distributed wavelengths covering the entire visible spectrum was used to represent the practical illumination. **(C)** A mosaic image of the USAF target through a sub-aperture in the central region on the lens array at ×3 magnification. Scale bar is 5 mm. **(D)** Sub-image of the USAF from **(C)**. Scale bar is 500 µm. **(E)** Result of the lateral resolution from the USAF imaging experiment. A total of 16 sub-apertures were imaged for each optical configuration. Error bars represent standard deviation. Paired t-tests were conducted between the ×5 group and all other magnification groups, *p* < 0.001 was denoted with * for comparison between 5× and 3×, 5× and 4×, and 5× and 6×. **(F)** A mosaic image of the µTUG seeded wellplate captured at 6× lens array configuration. The center of the pillar in each sub-image was tracked during “dummy” contraction. Scale bar is 5 mm. Error bar represents standard deviation. **(G)** SNR for the displacement tracked during the “dummy contraction” experiment. Data points were grouped by the tested displacements. For each data point, the specific displacement was travelled 10 times. Error bars represent standard deviation. Within each displacement group, paired *t*-tests were conducted between every magnification pair, *p* > 0.05 was denoted with ** for tests on 4× and 5×, 3× and 6× in both the 5 µm and 10 µm displacement groups. *p* > 0.05 was found for all magnification pairs in the 1 µm displacement group but was not denoted for the clarity of the figure.

The nominal numerical aperture (NA) of the optical system can be adjusted by changing the magnification of the lens array subsystem. In optical simulations performed on Zemax simulation, we found that, at 20% cutoff contrast, the modulation transfer function (MTF) reached the highest spatial frequency at 4× and 5× lens array magnification (Figure 3A). The point spread function (PSF) indicated that 4× had the widest first order Airy ring, followed by 5× and 6× (Figure 3B). Both analytic results implied the best optical configuration of our designed setup should use lens array between 4× and 5×.

The lateral image plane resolution was then experimentally tested by imaging a positive USAF 1951 high resolution target (#58-198, Edmund Optics). As shown in Figure 3C, the air force target was placed at the center of a sub-aperture. From each quadrant of the lens array, 4 sub-apertures were tested. The lateral resolution was obtained by justifying the least resolvable element on the air force target (Figure 3D). The same procedure was repeated for all four magnifications simulated before. Result validated that ×5 lens array magnification had a best lateral resolution of 10.08 ± 0.54 µm, agreed with the analytic values of ∼45 l p/mm and ∼10 µm in the MTF and PSF respectively (Figure 3E).

A second imaging experiment was performed using a µTUG-seeded wellplate without ECTs. The wellplate was fixed on 2-axis motorized sample stage (PLS-XY, Thorlabs). With a precision controller (encoder resolution 0.5 µm, feedback bandwidth 16 MHz; MCM3002, Thorlabs), the µTUGs were translated laterally in a sinusoidal pattern at 0.5 Hz to mimic the effect on micropillars of ECT beating (Figure 3F). Three levels of displacement, 1 μm, 5 μm, and 10 μm, were tested. A total of 10 translation experiments were recorded for each level of displacement. Using the previously described tracking program, time series of the µTUG displacement was plotted. From these plots, the signal-to-noise ratio (SNR) of the measured displacement was collected (Figure 3G). The best SNR recorded was above 200:1.

### 3.2 Chronic tracking of contractile forces

**FIGURE 4:**
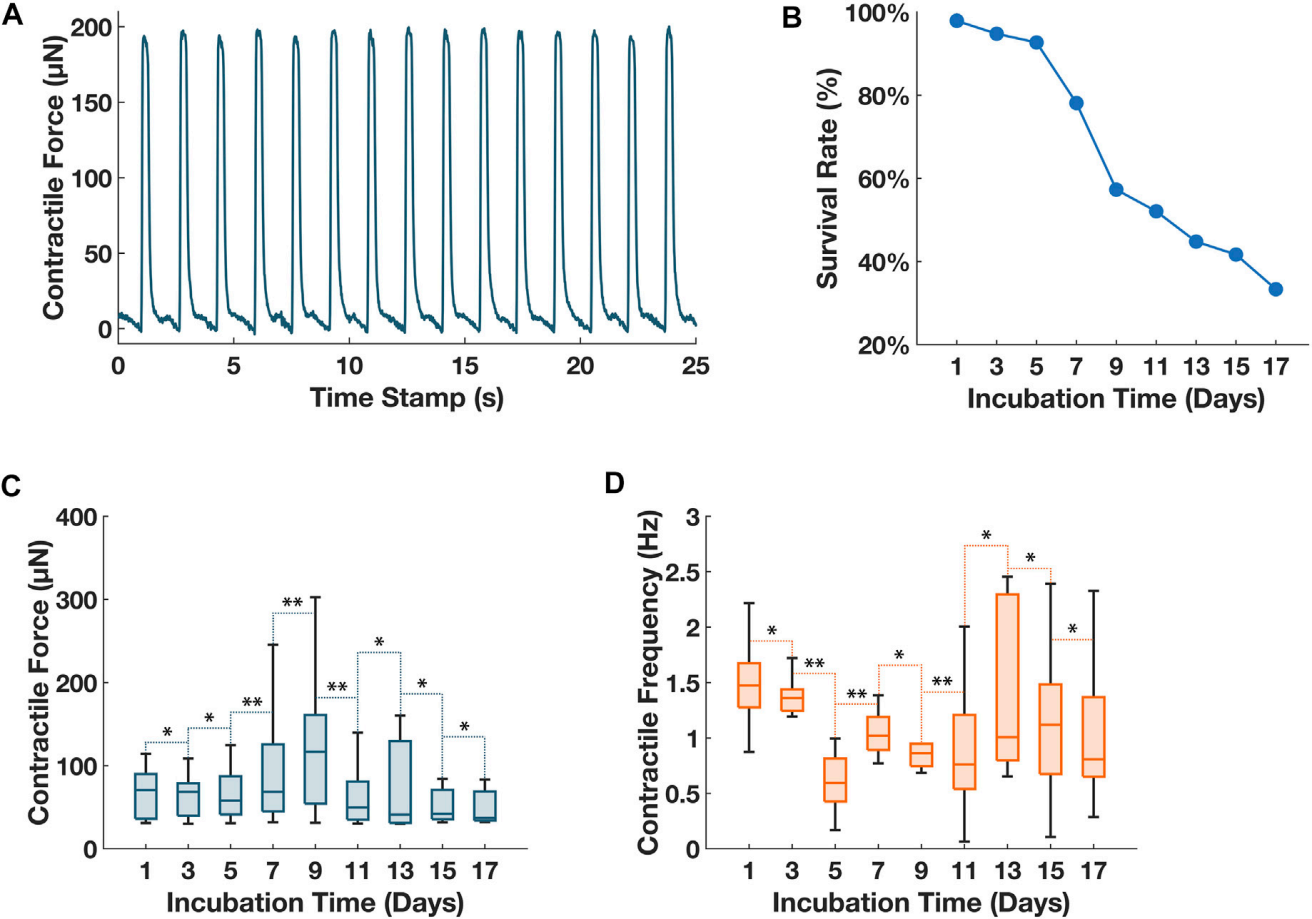
Chronic tracking of contractile forces for 96 ECTs seeded on a wellplate. **(A)** Sample plot of contractile force vs. time for ECT in well #4 on day 1. The imaged deflection was converted to contractile force using the experimental data of pillar stiffness of 5.3 μN/μm. **(B)** Tissue survival rate of 96 ECTs over the 17-day monitoring period. **(C)** Collective contractile force of all living ECTs on the 96-well plate over the 17-day monitoring period. **(D)** Collective contractile frequency of all living ECTs on the 96-well plate over the 17-day monitoring period. For **(C)** and **(D)**, vertical bars represent the extremes of each data group, horizontal bars within each box represent the medians of each data group. Whisker lines represent the minimum and maximum values for measurement of contractile amplitude and frequency for ECTs with an active spontaneous contraction on the respective dates. A balanced ANOVA test was performed between consecutive measurement days for both measurements of contractile amplitude and contractile frequency, *p* ≥ 0.01 between consecutive measurements was denoted as **, *p* < 0.01 between consecutive measurements was denoted as *.

With the validated optics, to satisfy the chronic tracking required by current cardiotoxicity screening protocols, we conducted repetitive contractile characterization of the ECTs over a prolonged period (17 days). A wellplate was molded with 96 µTUGs, we tested the µTUG stiffness from the used batch: measured µTUG stiffness was 5.30 ± 0.97 μN/μm (for a total of 20 samples). Then, the µTUGs were seeded with 3D ECTs. After compaction of ECTs, the wellplate was transferred into the stage top incubator of the imaging system. Starting on day 1, contractile force was measured every 48 h for an interval of 2000 frames (∼30 s). Peaks of the contractile force were averaged within each interval (Figure 4A). Culturing media was changed every 48 h from day 2. The wellplate was kept in the stage top incubator the entire duration of this test except for media change. Illumination was used only for the measurement period to avoid phototoxicity. The contractile force and the contractile frequency of all 96 ECTs were collected in Figures 4B–D.

### 3.3 Drug testing with isoproterenol

**FIGURE 5:**
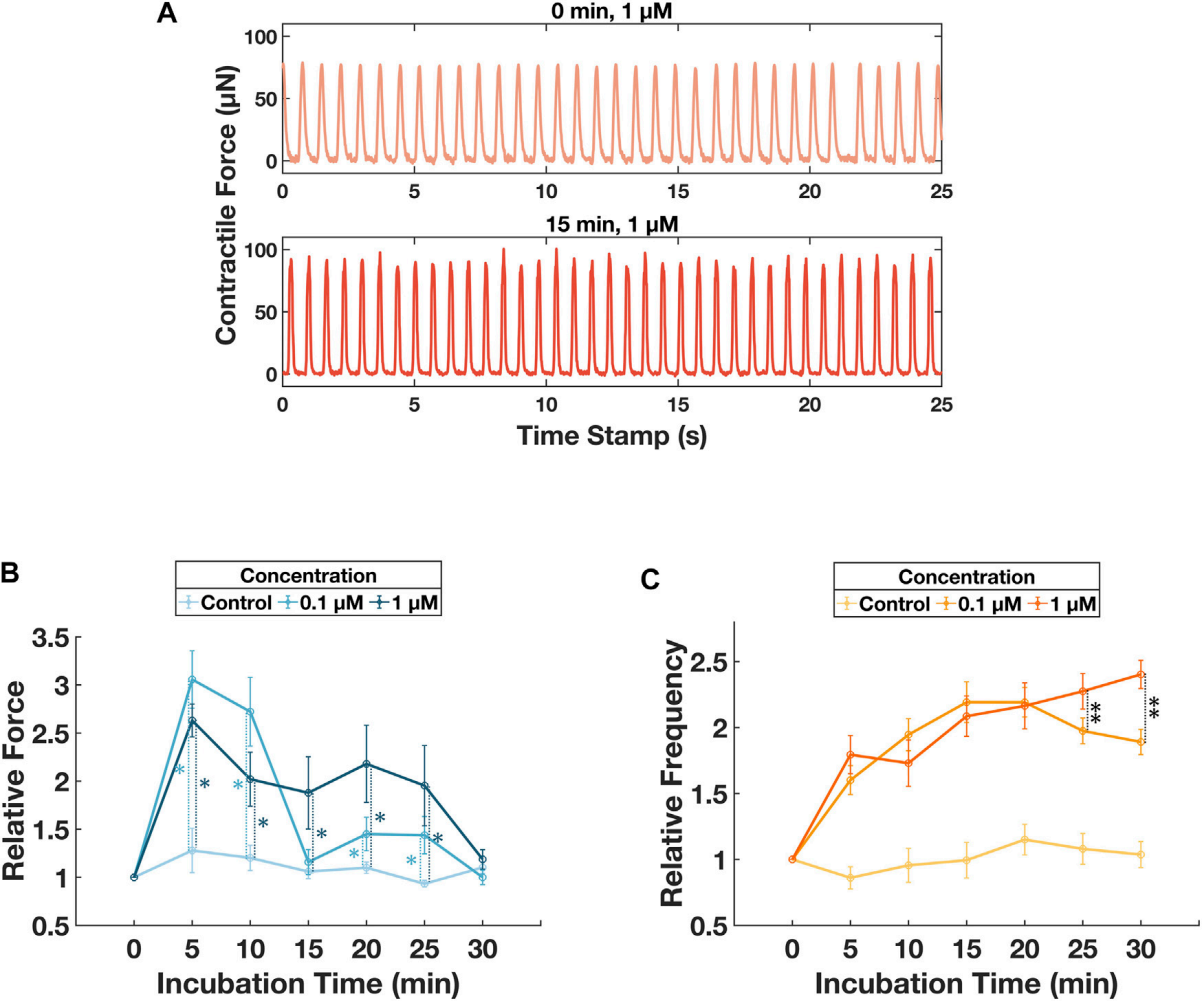
Monitoring of contractile behavior changes induced by different concentration of isoproterenol. **(A)** Sample force-time plots for ECT in well #14 on day 4 at 0 min and 15 min after culturing with 1 µM isoproterenol. **(B)** Tracking of the relative contractile force for 30 min after dosing with isoproterenol. Error bars represent standard deviations for the ECT groups dosed with respective concentrations. Unbalanced ANOVA tests were performed for concentration group 0.1 µM and 1 µM with respect to the control group. For concentration of 0.1 µM, *p* < 0.01, denoted as *, for incubation time of 5, 10, 20, and 25 min. For concentration of 1µM, *p* < 0.01, denoted as *, for incubation time of 5, 10, 15, 20, and 25 min. Additional unbalanced ANOVA tests were performed between concentration group of 0.1 µM and 1 µM, *p* < 0.01 for incubation time of 15, 20, and 25 min. **(C)** Tracking of the relative contractile frequency for 30min after dosing with isoproterenol. Error bars represent standard deviations for the ECT groups dosed with respective concentrations. Unbalanced ANOVA tests were performed for concentration group 0.1 µM and 1 µM with respect to the control group. For both concentration groups, *p* < 0.01, no denotation was used in the figure for the ease of viewing, for incubation time of 5, 10, 15, 20, 25, and 30 min. Additional unbalanced ANOVA tests were performed between concentration group of 0.1 µM and 1 µM, *p* < 0.01, denoted as **, for incubation time of 25 and 30 min.

A second ECT-seeded wellplate was administrated with drugs of known effects, positive inotrope isoproterenol was chosen for this experiment. Isoproterenol was diluted with the culturing media into two concentration groups with a control group (i.e., 0 μM, 0.1 µM, and 1 µM). Every 4 consecutive columns of the wellplate were designated for each concentration group. The isoproterenol media was distributed into a standard V-bottom 96-well plate and maintained at 37°C before adding to the ECTs. Started on day 1, ECT was nourished with normal media every 48 h. On day 4 and day 6, before adding the compound, the sample plate was imaged for a reference contractile force measurement. A 96-well liquid handler with environmental control block (VIAFLO96, INTEGRA) was used to dose the compound-media mix into the ECT seeded wellplate while maintaining the culture environment. The wellplate was transferred to the imaging system immediately. Acquisition was triggered every 5 min for 30 min. Finally, the ECTs were washed with PBS solution and loaded with drug-free culturing media. Day 4 and day 6 data are combined and presented in Figure 5.

## 4 Discussion

We have presented a label-free, wide-field imaging system that measures contractile activities for 3D ECTs seeded on a 96-well plate. In addition to satisfying the imaging requirement, we have successfully scaled up the manufacturing of PDMS µTUG scaffolds to the 96-well format. An automated image acquisition, processing and data illustration pipeline was scripted in MATLAB. Each module ran at > 60 Hz, enabling video rate contractile force monitoring. Compared to other current video microscopy imaging techniques for 3D ECTs (Mannhardt et al., 2016; Thavandiran et al., 2020; Gracioso Martins et al., 2021), our system is the first of its kind to show capability of true 96-well tissue seeding and parallel contractile force sensing, strongly aligning with the technical need and market pull for high throughput toxicity screening with 3D ECTs.

Functionality of our system was validated by two experiments: continuous culturing and monitoring of 96 ECTs over 17 days; and contractile responses to isoproterenol. The experimental settings were compliant with current low throughput cardiotoxicity assay (Zhao et al., 2017; Ronaldson-Bouchard et al., 2018; Gintant et al., 2019). The contractile force and frequency responses matched with reported interaction between ECTs and isoproterenol (Feric et al., 2019; Goldfracht et al., 2019; Takada et al., 2022). Our experiments with multiple compound concentrations distributed across well plates were conceived as one way to demonstrate the utility of multiplexing on the platform. There are no logistical barriers to extending that multiplexing to administering different compounds at different concentrations. Additional characterization of these tissues after contractility measurements was not done. However, ECTs of this type and in these scaffolds have been characterized previously. (Legant et al., 2009; Boudou et al., 2012; Hinson et al., 2015; Zhang et al., 2021; Das et al., 2022).

Given the functional imaging resolution limit of 10 μm, localization precision of 1 μm, and a stiffness of µTUG scaffold of 5.3 μN/μm, the resultant nominal sensitivity of contractile amplitude for the described parallel high throughput imaging system is 0.2 µN, and the system can measure contractile forces with relatively high SNR (∼200:1).

The temporal resolution of the system is limited primarily by the frame rate of imaging, nominally 60 Hz. Current regulatory guidelines are based on screening drug-induced action potential prolongation of 5–10 ms, which is smaller than the 33 ms Nyquist-limited temporal resolution achievable using a 60 Hz frame rate. To reach the frame rates relevant for that task, the camera frame rate could be increased further by binning 2 × 2 or 4 × 4 pixels. The current system is oversampled by almost a factor of three by the camera, which features 3.45 µm pixels in an optical system with ∼10 µm resolution. We could achieve a frame rate of 133 Hz (15 ms temporal resolution) with no loss of image quality if we used 2 × 2 pixel binning, and a frame rate of 200 Hz (10 ms temporal resolution) with modest reduction in lateral resolution if we used 4 × 4 pixel binning. Prior studies of action potential dynamics following drug introduction in tissue monolayers used imaging frame rates of 200 Hz (Herron et al., 2016) in an individual ECT assay. In calcium imaging, dozens of drug-induced proarrhythmic risks were assessed using a standardized protocol at three test sites, with imaging frame rates of 62.5 Hz (Lu et al., 2019). As this is comparable to the frame rate in the experiments reported here, we believe that our frame rate is already compatible with requirements for calcium imaging.

The current study did not include imaging of fluorescence-based electrophysiological dyes, but the system was designed to accommodate fluorescence imaging with minor modifications, namely, installing a commercially available 96 well plate transillumination source (e.g., Lumos 96, 475 nm wavelength, 3900 W/m^2^, Axion Biosystems Inc.), and adding an emission filter to the camera sensor. It would be speculative to predict performance of such a system, though we can estimate whether these two modifications could enable fluorescence membrane potential sensing using our optical system. The illumination source cited would produce a maximum photon flux I at the sample of 9.3 × 10^21^ photons/(m^2^s). For a common membrane sensor (FluoVolt Thermo Fisher Scientific Inc.) the surface density S of fluorophores is of order 10^17^ molecules/m^2^, the absorption cross section σ_a_ is 9.6 × 10^−21^ m^2^/molecule, and the quantum yield F is 0.057 (Deal et al., 2020; Miller et al., 2012, in Supplementary Material). The fluorescence output is the product ISσ_a_F, or 5.1 × 10^17^ photons/(m^2^s). The total tissue cross sectional area for the 96 ECTs in a well plate is about 1.0 × 10^−4^ m^2^ meaning that 5.1 × 10^13^ photons/s of emitted fluorescence F emanates from the well plate. The numerical aperture of the detection system is 0.056, which leads to a light collection efficiency η of 7.8 × 10^−4^ at the camera sensor. The camera spectral response R_s_ is 0.85 and its quantum efficiency Q_e_ is 0.62. So, total fluorescence reaching the camera sensor is R_s_ Q_e_ η F, or 2.1 × 10^10^ photons/sec. The magnified ECT surface in the mosaic image represents about 20% of the camera sensor’s pixels. If the fluorescence were distributed evenly across those 2.4 × 10^6^ pixels, the fluorescence on each pixel would be 8,800 photons/(pixel s), several orders of magnitude larger than the sensor’s detection threshold of 4.6 photons/(pixel s). This rough analysis ignores photobleaching and other effects that might impact imaging performance. Nevertheless, it suggests that membrane potential imaging might be possible with only minor changes to the system.

The other functionality needed for electrophysiological characterization is the means to electrically stimulate the tissues. Prototype of a 96-well electrode array and a high power electrical controller have been built. Upon future validation, our optical innovation will allow imaging of both the contractile and electrophysiological responses of the ECT within one single system.

More broadly, any functional assay of engineered tissues—not limited to ECTs—with similar optical requirements in terms of resolution, FOV and speed can be performed with our optical system. A few candidates we have identified include neuron excitation imaging and endothelial cells and fibroblasts dynamics in wound closure (Dana et al., 2019; Das et al., 2022; Reilly et al., 2022).

In conclusion, we have demonstrated a high-throughput screening system for contractile force evaluation. Preliminary drug tests have shown the potential of this system to fit FDA’s framework of cardiotoxicity screening with 3D ECTs. With future inclusion of multiple modes of functional characterization for ECTs, and expansion to other engineered tissue types, this platform has potential for translation and widespread use in high-throughput characterization of engineered tissues.

## Data Availability

The original contributions presented in the study are included in the article/Supplementary Material, further inquiries can be directed to the corresponding authors.
