# Supplementary material for: High throughput screening system for engineered cardiac tissues
Source: Front Bioeng Biotechnol. 2023 May 11;11:1177688. doi: 10.3389/fbioe.2023.1177688 (PMC10210164; doi:10.3389/fbioe.2023.1177688)
Supplement: Supplementary file 1 [file DataSheet1.docx]

Supplementary Material

High Throughput Screening System for Engineered Cardiac Tissues

Marshall S. Ma^1,2†^, Subramanian Sundaram^3†^, Lihua Lou^4†^, Arvind Agarwal^4*^, Christopher S. Chen^3*^ and Thomas G. Bifano^1,2*^

*** Correspondence:** Arvind Agarwal: [agarwala@fiu.edu](mailto:agarwala@fiu.edu); Christopher S. Chen: [chencs@bu.edu](mailto:chencs@bu.edu); Thomas G. Bifano: [tgb@bu.edu](mailto:tgb@bu.edu)

# Supplementary Data

Not applicable.

# Supplementary Figures and Tables

## Supplementary Figures

**Supplementary Figure 1.** Design specification of the lens array.

**Supplementary Figure 2.** Design specification of the parallel stamping mold.
